# Retrospective cohort study to determine the effect of preinjury antiplatelet or anticoagulant therapy on mortality in patients with major trauma

**DOI:** 10.3389/fmed.2022.1089219

**Published:** 2023-01-09

**Authors:** Fuminori Yamaji, Hideshi Okada, Ryo Kamidani, Yuki Kawasaki, Genki Yoshimura, Yosuke Mizuno, Yuichiro Kitagawa, Tetsuya Fukuta, Takuma Ishihara, Kodai Suzuki, Takahito Miyake, Norihide Kanda, Tomoaki Doi, Takahiro Yoshida, Shozo Yoshida, Shinji Ogura

**Affiliations:** ^1^Advanced Critical Care Center, Gifu University Hospital, Gifu, Japan; ^2^Abuse Prevention Center, Gifu University Graduate School of Medicine, Gifu, Japan; ^3^Innovative and Clinical Research Promotion Center, Gifu University Hospital, Gifu, Japan

**Keywords:** trauma, antiplatelet therapy, anticoagulant therapy, cohort study, J-OCTET 2, injury

## Abstract

**Objective:**

This study aimed to compare outcomes among patients who sustained major trauma from injury with and without receiving antiplatelet therapy (APT) or anticoagulant therapy (ACT) to test the hypothesis that APT does not increase the risk of mortality. However, ACT increases the mortality risk in the acute phase of trauma.

**Methods:**

Patients registered in the Japanese Observational body for Coagulation and Thrombolysis in Early Trauma 2 between April 2017 and March 2018 who had sustained a severe injury in any anatomic region of the body, as determined using an injury severity score (ISS) ≥ 16 were included in this retrospective cohort study. We analyzed the mortality within 24 h from the arrival using a multivariable linear regression analysis adjusted for several confounding variables.

**Results:**

We identified 1,186 eligible participants who met the inclusion criteria for this study: 105 in the APT (cases), 1,081 in the non-antiplatelet therapy (nAPT) group (controls), 65 in the ACT (cases), and 1,121 in the non-anticoagulant therapy (nACT) group (controls). The mortality within 24 h in the ACT group was significantly higher than in the nACT group (odds ratio 4.5; 95%CI: 1.2–16.79; *p* = 0.025); however, there was no significant difference between the two groups with or without the antiplatelet drug (odds ratio 0.32; 95%CI: 0.04–2.79; *p* = 0.3) administration. Other outcomes, like the 28-day mortality, mortality at discharge, and surgery for hemostasis, were not significantly different between regular users and non-users of either antiplatelet or anticoagulant drugs.

**Conclusion:**

Regular antiplatelet medications did not increase mortality within 24 h, 28 days, or at discharge in patients with major trauma, suggesting that standard treatment, including surgery, is sufficient.

## 1. Introduction

A quarter to half of the patients with major trauma in some countries are over the age of 60 years due to an aging society, particularly in the West and Japan (1–3). Elderly patients with trauma are different from younger patients in residual physiological functions, the complexity of comorbidities, types of regular medications, and mechanisms of injury (1, 2).

Previous studies have shown that starting antiplatelet therapy (APT) before injury significantly increases mortality risk and unfavorable outcomes in patients with traumatic brain injury (TBI) (3–6). However, some studies suggest no link between APT and increased mortality in TBI (7–9). To the best of our knowledge, it is unclear how APT before injury affects overall trauma mortality. Therefore, to test the hypothesis that pre-injury APT does not increase the risk of mortality; however, anticoagulant therapy (ACT) increases it in the acute phase of trauma, this study aimed to compare outcomes among injured patients with major trauma with and without administration of APT or ACT.

## 2. Materials and methods

### 2.1. Study oversight and design

We conducted a retrospective cohort study between April 2017 and March 2018 using the Japanese Observational study for Coagulation and Thrombolysis in Early Trauma 2 (J-OCTET 2) during the observation period. Tohoku University institutional research ethics committee approved the use of the J-OCTET 2 (approval #2020-1-898, approved on January 15, 2021). Furthermore, the Gifu University institutional ethics committee approved this study (approval #2022-141, approved on October 12, 2022). In addition, the institutional ethics committees of Gifu University Graduate School of Medicine approved the substitution of an opt-out notice of informed consent from patients due to the retrospective nature of the study, whose design was based on computerized data with anonymous selection.

### 2.2. Study patients

Overall, data from 1,213 patients with trauma were registered in the J-OCTET 2 between April 2017 and March 2018. They had an injury severity score (ISS) ≥ 16, indicating a severe injury in any region. The following were the exclusion criteria: (1) cases in which consent to participate was not obtained, (2) time of injury was unclear, (3) the patient was transferred from a different hospital, (4) declined active treatment, (5) had a cardiopulmonary arrest on arrival, (6) had a burn injury, and (7) pregnancy, or had coexisting cirrhosis of the liver in the J-OCTET 2. Additionally, we excluded cases with missing data on oral medications from our analysis.

### 2.3. Data collection

We collected the following data from the electronic medical records: age, sex, ISS, time from accident to hospital arrival, drug history, Charlson Risk Index, systolic blood pressure (SBP) on arrival, heart rate (HR) on arrival, the respiratory rate on arrival, Glasgow coma scale score, lactate level, fibrinogen (Fbg) level, hemoglobin (Hb) level, platelet level, Focused Assessment with Sonography for Trauma (FAST), and prehospital care. The six FAST search sites are the pericardiac cavity, bilateral thoracic cavity, Morrison fossa, perisplenic fossa, and Douglas fossa. There was no predefined transfusion protocol in this study, including emergency reversal of acute major bleeds in patients on ACT, which was based on the physician’s clinical judgment.

### 2.4. Primary and secondary outcomes

The primary outcome of interest in this study was the mortality within 24 h of arrival. The secondary outcomes included 28 day-mortality, mortality at hospital discharge, surgical hemostatic intervention, transcatheter arterial embolization, and transfusion requirement within 24 h. The transfusion requirement was tabulated and analyzed separately for red blood cells, fresh frozen plasma (FFP), and platelet concentrate (PC).

### 2.5. Sample size

The sample size in this study was determined based on the number of covariates included in the statistical model for the primary analysis and overfitting (10) and based on data availability.

### 2.6. Statistical analysis

Continuous data were described using the median and interquartile range, and categorical data were described using frequencies with proportions. To evaluate the effect of regular use of anticoagulants and antiplatelet drugs on accidental trauma, we conducted a multivariable linear regression analysis adjusted for covariates, including age, ISS, time from accident to hospital arrival, Charlson Risk Index, SBP, HR, RR, lactate, Glasgow coma scale score, on arrival. FFP and PC were included as covariates only in analyses whereby the mortality within 24 h of arrival, 28-day mortality, and mortality at hospital discharge were the objective variables. The covariates were selected as potential confounders *a priori* based on previous studies (11) and expert advice from a physician in the field. The number of covariates was restricted enough to avoid overfitting. The degree of overfitting of the regression model was confirmed by the optimal parameter obtained from the calibration plots from 150 bootstrap validations. Based on the optimal parameter < 0.2, the model was determined not to be overfitting. We also evaluated the effects of the antiplatelet drug or anticoagulant use on the secondary outcomes. Binary outcomes were evaluated similarly using a multivariable logistic regression model as in the primary analysis. For continuous outcomes without normality, the multivariable proportional odds logistic regression model was used to evaluate the association with the antiplatelet drug or anticoagulant use. Proportional odds logistic regression, also known as ordinal logistic regression, is a popular model for ordinal categorical outcome variables, which also works well for skewed continuous outcomes using ranks of data. A two-sided *p*-value < 0.05 was considered statistically significant. All analyses were performed using R software (version 4.2.1; available at http://www.r-project.org) (12).

## 3. Result

### 3.1. Baseline characteristics

Overall, 1,213 patients were enrolled between April 2017 and March 2018. The analysis included 1,186 eligible participants who met the criteria of this study: 105 in the APT (cases), 1,081 in the non-antiplatelet therapy (nAPT) group (controls), 65 in the ACT (cases), and 1,121 in the non-anticoagulant therapy (nACT) group (controls). Table 1 summarizes the clinical characteristics of the patients (Table 1). There were no significant differences in sex, time from accident to hospital arrival, Glasgow coma scale score at arrival, or FAST findings at any site between the two groups with or without antiplatelet use. Age, Charlson Risk Index, SBP, and Fbg level were higher in the APT group than in the control group. ISS, HR, RR, lactate level, Hb level, and platelet counts were lower in the APT group than in the control group. There were no significant differences in sex, ISS, SBP, Glasgow coma score, or FAST findings at any site between the two groups with or without anticoagulant use. Age, time from accident to hospital arrival, Charlson risk index, and Fbg level was higher in the ACT group than in the control group. HR, RR, Lac level, Hb level, and platelet counts were lower in the ACT group than in the control group.

**TABLE 1:** Clinical characteristics of the participants.

			Using of antiplatelet drugs			Using of anticoagulant		
Variable	*N*	Overall, *N* = 1,186^a^	No, *N* = 1,081^a^	Yes, *N* = 105^a^	*P*-value^b^	No, *N* = 1,121^a^	Yes, *N* = 65^a^	*P*-value^b^
Age	1,186	66 (47, 77)	64 (45, 76)	79 (70, 84)	<0.001	64 (46, 76)	80 (71, 85)	<0.001
Sex	1,186				0.735			0.325
Female		338 (28.5%)	310 (28.7%)	28 (26.7%)		316 (28.2%)	22 (33.8%)	
Male		848 (71.5%)	771 (71.3%)	77 (73.3%)		805 (71.8%)	43 (66.2%)	
ISS	1,186	22.0 (17.0, 29.0)	22.0 (17.0, 29.0)	20.0 (16.0, 25.0)	0.025	22.0 (17.0, 29.0)	25.0 (17.0, 25.0)	0.588
Time_from_accident_to_hospital_arrival	1,170	44.0 (33.0, 61.0)	44.0 (33.0, 60.0)	46.0 (35.0, 63.8)	0.330	44.0 (33.0, 60.0)	52.0 (36.5, 80.5)	0.003
Charlson risk index	1,040	0.0 (0.0, 1.0)	0.0 (0.0, 0.0)	2.0 (1.0, 3.0)	<0.001	0.0 (0.0, 1.0)	2.0 (0.8, 2.0)	<0.001
SBP_on_arrival	1,178	134.0 (110.0, 156.8)	132.0 (110.0, 155.0)	148.0 (123.0, 174.0)	<0.001	133.0 (110.0, 156.0)	140.0 (120.0, 158.0)	0.244
HR_on_arrival	1,185	84.0 (72.0, 100.0)	85.0 (72.0, 101.0)	78.0 (69.0, 90.0)	0.001	84.5 (72.0, 101.0)	79.0 (68.0, 90.0)	0.008
RR_on_arrival	1,179	20.0 (17.0, 24.0)	20.0 (17.2, 24.0)	18.0 (16.0, 23.0)	0.016	20.0 (17.0, 24.0)	18.0 (16.0, 21.0)	0.025
GCS_on_arrival	1,186	14.0 (10.0, 15.0)	14.0 (10.0, 15.0)	14.0 (12.0, 15.0)	0.717	14.0 (10.0, 15.0)	14.0 (12.0, 15.0)	0.68
Lactate_on_arrival	1,054	2.4 (1.7, 3.7)	2.5 (1.7, 3.9)	1.6 (1.2, 2.2)	<0.001	2.5 (1.7, 3.8)	1.7 (1.5, 2.2)	<0.001
Fbg_on_arrival_2	1,007	241.0 (191.0, 290.0)	238.0 (185.0, 283.0)	287.0 (226.8, 337.0)	<0.001	239.0 (188.2, 286.0)	276.0 (244.0, 333.0)	<0.001
Hb_on_arrival	1,181	13.1 (11.7, 14.5)	13.2 (11.8, 14.6)	12.5 (11.2, 13.7)	<0.001	13.2 (11.8, 14.5)	11.6 (10.7, 13.2)	<0.001
Plt_on_arrival	1,180	21.4 (17.1, 26.0)	21.6 (17.4, 26.4)	18.4 (14.8, 22.2)	<0.001	21.6 (17.4, 26.3)	17.9 (14.1, 20.9)	<0.001
FAST (pericardiac cavity)	1,024				0.211			0.300
Negative		1,004 (98.0%)	925 (98.2%)	97 (96.3%)		950 (98.1%)	54 (96.4%)	
Positive		20 (2.0%)	17 (1.8%)	3 (3.7%)		18 (1.9%)	2 (3.6%)	
FAST (Lt. thoracic cavity)	1,026				0.659			0.619
Negative		1,007 (98.1%)	927 (98.2%)	80 (97.6%)		951 (98.0%)	56 (100.0%)	
Positive		19 (1.9%)	17 (1.8%)	2 (2.4%)		19 (2.0%)	0 (0.0%)	
FAST (Rt. thoracic cavity)	1,026				>0.999			>0.999
Negative		1,010 (98.4%)	929 (98.4%)	81 (98.8%)		954 (98.4%)	56 (100.0%)	
Positive		16 (1.6%)	15 (1.6%)	1 (1.2%)		16 (1.6%)	0 (0.0%)	
y FAST (Morrison fossa)	1,025				0.250			0.166
Negative		981 (95.7%)	900 (95.4%)	82 (98.8%)		925 (95.5%)	56 (100.0%)	
Positive		44 (4.3%)	43 (4.6%)	1 (1.2%)		44 (4.5%)	0 (0.0%)	
FAST (perisplenic cavity)	1,025				0.160			0.394
Negative		998 (97.4%)	916 (97.1%)	82 (100.0%)		942 (97.2%)	56 (100.0%)	
Positive		27 (2.6%)	27 (2.9%)	0 (0.0%)		27 (2.8%)	0 (0.0%)	
FAST (Douglas fossa)	1,025				0.715			0.649
Negative		999 (97.5%)	918 (97.3%)	81 (98.8%)		945 (97.5%)	54 (96.4%)	
Positive		26 (2.5%)	25 (2.7%)	1 (1.2%)		24 (2.5%)	2 (3.6%)	
Pre_hospital_care	1,185	235 (19.8%)	219 (20.3%)	16 (15.2%)	0.249	221 (19.7%)	14 (21.9%)	0.632

^a^Median (IQR); *n* (%). ^b^Wilcoxon rank sum test; Fisher’s exact test. ISS, injury_severity_score; SBP, systolic blood pressure; HR, heart rate; RR, respiratory rate; GCS, Glasgow coma scale; Fbg, fibrinogen; Hb, hemoglobin; Plt, platelet count; FAST, Focused Assessment with Sonography for Trauma.

### 3.2. Outcomes

#### 3.2.1. Primary outcomes

Table 2 presents the outcome variables of our study. The mortality rate within 24 h was 2.9–7.7%, and the overall mortality was 5.6% (66 of 1,186 patients). The mortality within 24 h in the ACT group was significantly higher compared to the nACT group (odds ratio 4.5; 95%CI: 1.2–16.79; *p* = 0.02); however, there was no significant difference between the two groups with or without antiplatelet drug administration (odds ratio 0.32; 95%CI: 0.04–2.79; *p* = 0.3).

**TABLE 2:** Multivariable binary/proportional odds logistic analysis.

			Using of antiplatelet drugs				Using of anticoagulant			
Outcome	N	Overall, *N* = 1,186	No, *N* = 1,081^a^	Yes, *N* = 105^a^	Odds ratio (95% CI)	*P*-value^b^	No, *N* = 1,121^a^	Yes, *N* = 65^a^	Odds ratio (95% CI)	*P*-value^b^
Mortality within 24 h	1,185				0.32 (0.04, 2.79)	0.3			4.5 (1.2, 16.79)	0.025
Died		66 (5.6%)	63 (5.8%)	3 (2.9%)			61 (5.4%)	5 (7.7%)		
Survived		1,119 (94.4%)	1,017 (94.2%)	102 (97.1%)			1,059 (94.6%)	60 (92.3%)		
28 day-mortality	1,164				0.63 (0.21, 1.89)	0.406			1.2 (0.42, 3.47)	0.736
Died		114 (9.8%)	104 (9.8%)	10 (9.5%)			107 (9.7%)	7 (10.8%)		
Survived		1,050 (90.2%)	955 (90.2%)	95 (90.5%)			992 (90.3%)	58 (89.2%)		
Mortality at hospital discharge	1,182				0.49 (0.16, 1.52)	0.216			1.05 (0.36, 3.09)	0.927
Died		129 (10.9%)	118 (11.0%)	11 (10.5%)			120 (10.7%)	9 (13.8%)		
Survived		1,053 (89.1%)	959 (89.0%)	94 (89.5%)			997 (89.3%)	56 (86.2%)		
Surgery for hemostasis	1,183	190 (16.1%)	175 (16.2%)	15 (14.3%)	1.66 (0.74, 3.7)	0.215	181 (16.2%)	9 (14.1%)	0.79 (0.30, 2.12)	0.644
TAE	1,184	148 (12.5%)	138 (12.8%)	10 (9.5%)	1.52 (0.64, 3.60)	0.341	147 (13.1%)	1 (1.6%)	0.08 (0.01, 0.64)	0.018
RBC transfusion	1,172	0.0 (0.0, 560.0)	0.0 (0.0, 280.0)	0.0 (0.0, 280.0)	1.59 (0.86, 2.95)	0.139	0.0 (0.0, 560.0)	0.0 (0.0, 0.0)	0.34 (0.15, 0.78)	0.011
FFP transfusion	1,171	0 (0.0, 480.0)	0.0 (0.0, 720.0)	0.0 (0.0, 360.0)	2.22 (1.22, 4.05)	0.009	0.0 (0.0, 720.0)	0.0 (0.0, 0.0)	0.37 (0.17, 0.82)	0.014
PC transfusion	1,167	0.0 (0.0, 0.0)	0.0 (0.0, 0.0)	0.0 (0.0, 0.0)	3.16 (1.55, 6.42)	0.002	0.0 (0.0, 0.0)	0.0 (0.0, 0.0)	0.74 (0.3, 1.81)	0.505

^a^Statistical data are presented as the median (interquartile range) or *n* (%). ^b^Statistical tests performed: multivariable logistic regression; multivariable proportional odds logistic regression. TAE, transcatheter arterial embolization; RBC, red blood cell; FFP, fresh frozen plasma; PC, platelet concentrate.

#### 3.2.2. Secondary outcomes

There were no significant differences in the 28-day mortality, mortality at discharge, or surgery for hemostasis between regular users and non-users of either antiplatelet or anticoagulant drugs. The number of patients who received TAE was 147 (13.1%) and 1 (1.6%) in the nACT and ACT groups, respectively; the rate of TAE was significantly lower in the ACT group with an odds ratio of 0.08; 95%CI: 0.01–0.64; *p* = 0.018. For transfusion volume, RBC transfusion was significantly lower in the ACT group (odds ratio 0.34; 95%CI: 0.15–0.78; *p* = 0.011), whereas FFP and PC transfusions were significantly higher in the APT group (FFP; odds ratio 2.22; 95%CI: 1.22–4.05; *p* = 0.009 and PC; odds ratio 3.16; 95%CI: 1.55–6.42; *p* = 0.002).

#### 3.2.3. Sub-group analysis

Additionally, a sub-group analysis excluding TBI with an abbreviated injury scale of three or more points was performed (Table 3). After excluding TBI with an abbreviated injury scale of three or more points, 519 patients were remaining, with 36 administered APT and 15 with ACT. However, they survived for 24 h in both cases. Overall mortality rates within 24 h, 28-day mortality, and mortality at hospital discharge were 2.5, 4.1, and 4.8%, respectively.

**TABLE 3:** Outcome variables excluding TBI with abbreviated injury scale of 3 or more points.

			Using of antiplatelet drugs		Using of anticoagulant	
Outcome	*N*	Overall, *N* = 519	No, *N* = 483^a^	Yes, *N* = 36^a^	No, *N* = 504^a^	Yes, *N* = 15^a^
Mortality within 24 h	518					
Died		13 (2.5%)	13 (2.7%)	0 (0.0%)	13 (2.6%)	0 (0.0%)
Survived		505 (97.5%)	469 (97.3%)	36 (100.0%)	490 (97.4%)	15 (100.0%)
28 day-mortality	508					
Died		21 (4.1%)	19 (4.0%)	2 (5.6%)	21 (4.3%)	0 (0.0%)
Survived		487 (95.9%)	453 (96.0%)	34 (94.4%)	472 (95.7%)	15 (100.0%)
Mortality at hospital discharge	517					
Died		25 (4.8%)	23 (4.8%)	2 (5.6%)	25 (5.0%)	0 (0.0%)
Survived		492 (95.2%)	458 (95.2%)	34 (94.4%)	477 (95.0%)	15 (100.0%)
Surgery for hemostasis	517	82 (15.9%)	79 (16.4%)	3 (8.3%)	80 (15.9%)	2 (13.3%)
TAE	518	85 (16.4%)	79 (16.4%)	6 (16.7%)	85 (16.9%)	0 (0.0%)
RBC transfusion	510	0.0 (0.0, 560.0)	0.0 (0.0, 560.0)	0.0 (0.0, 420.0)	0.0 (0.0, 560.0)	0.0 (0.0, 0.0)
FFP transfusion	510	0 (0.0, 480.0)	0.0 (0.0, 720.0)	0.0 (0.0, 360.0)	0.0 (0.0, 600.0)	0.0 (0.0, 0.0)
PC transfusion	508	0.0 (0.0, 0.0)	0.0 (0.0, 0.0)	0.0 (0.0, 0.0)	0.0 (0.0, 0.0)	0.0 (0.0, 0.0)

^a^Statistical data are presented as the median (interquartile range) or n (%). TAE, transcatheter arterial embolization; RBC, red blood cell; FFP, fresh frozen plasma; PC, platelet concentrate.

## 4. Discussion

This study highlights the regular use of antiplatelet drugs did not increase mortality within 24 h, 28-day mortality, or mortality at hospital discharge; however, the use of anticoagulants increased mortality within 24 h. Furthermore, it suggests that standard treatment, including surgery, is sufficient even when treating patients with trauma who are regularly administered antiplatelet drugs.

Geriatric trauma has been increasingly common in the Western and Japan, related to population aging. In the UK, more than a quarter of patients with trauma are over 75 years old, which has obvious implications for national and local healthcare planning, particularly for Major Trauma Centers and Emergency Departments (13). It has been reported that the percentage of patients with geriatric trauma has increased, with 47.8% of the Dutch Trauma Registry being over 65 years old in 2014 and 52.9% of the Japanese Nationwide Trauma Registry being over 60 years old between 2004 and 2015 (14, 15). Several studies reported the risk of mortality is 2.5–5.6 times higher in patients with geriatric trauma (16–19). It was reported that the mechanisms and patterns of injury among elderly patients differ from those among younger individuals. The most common site of injury in the elderly is the extremity, and often ground-level falls rather than high-energy trauma; however, age can be an independent risk factor for mortality (20). Thus, in geriatric trauma, even low-energy trauma often leads to severe trauma due to original physical vulnerability, involvement of comorbidities, and current oral medications that negatively affect pathophysiology and treatment.

Recently, the number of elderly patients with trauma who must be administered anticoagulant or antiplatelet drugs due to cardiovascular and cerebrovascular diseases or genetic diseases has been gradually increasing (21–23). Preinjury ACT has long been found to influence mortality and unfavorable outcomes significantly. Lee et al. reported that preinjury ACT was associated with a higher risk of overall mortality (OR 2.12, 95%CI 1.79–2.51, *p* < 0.00001), in-hospital mortality (OR 2.04, 95%CI 1.66–2.52, *p* < 0.00001), intracranial hemorrhage (OD 1.99, 95%CI 1.61–2.45, *p* < 0.00001), and shorter length of hospital stay (MD 0.50, 95%CI 0.03–0.97, *p* = 0.04) in a systematic review and meta-analysis (24). Brain tissue injury stimulates the tissue factor pathway of coagulation in blunt TBI, resulting in various degrees of systemic bleeding tendency and coagulopathy (25–27). In our study, patients who were administered antithrombotic drugs with anticoagulant and antiplatelet before injury were significantly older; nevertheless, only patients administered ACT had significantly elevated mortality. Although the registry study does not allow for a detailed examination, the extremely low rate of TAE in the ACT group suggested that the patients may have had trauma requiring surgery for hemostasis in relative terms, or they may have had multiple bleeds non-amenable to TAE. There was no significant difference in the 28 day-mortality or mortality at hospital discharge in the ACT group, however, since the half-life of anticoagulants, especially DOACs, is at most 12 h, the effect of pre-injury medication was minor, and since the prognosis of severe trauma itself can be affected by definitive treatment during the so-called “golden hour,” only early mortality was considered significant. Therefore, there may be justification for discontinuing anticoagulant drug administration when patients who are administered ACT suffer major trauma.

In contrast, Yuval et al. reported that antithrombotic drugs such as anticoagulants and antiplatelet drugs did not significantly increase mortality or blood transfusion requirements among patients with major trauma, including patients with head trauma (28). Thus, the efficacy of APT before the injury and continued administration of antiplatelet drugs after injury remains controversial. Initially, it was reported that discontinuing APT administration increased the risk of thromboembolism significantly, especially in patients with coronary heart disease. Moreover, the risk of coronary thrombosis after withdrawal of APT is greater than the risk of surgical bleeding (29). However, the risk of stroke is relatively low, with only approximately 2% occurring within 30 days of APT discontinuation (30).

Several reports revealed that APT before the injury was significantly associated with increased mortality and unfavorable outcomes in blunt TBI (4–6). In a systemic review and meta-analysis, Batchelor et al. reported a slightly increased risk of death in patients administered APT with blunt TBI (3). Others have reported an increased need for surgery, higher hospitalization rates, and poor discharge status in moderate patients with head trauma administered APT (7). Jones et al. also reported a high incidence of intracranial rebleeding episodes in similar situations (31). Conversely, several studies revealed no significant association between mortality due to APT and head injury (7, 8). To exclude the effect of coagulopathy induced by TBI on the outcomes, patients with concomitant TBI with an abbreviated injury scale of 3 or more points were excluded from the subgroup analysis. However, the extremely low mortality rates in both the APT and ACT groups precluded statistical analysis, and the effect of TBI on outcomes could not be determined in this study.

There is no clear evidence of the effect of preinjury APT on mortality and other outcomes, particularly for trauma other than single TBI. Furthermore, the ability of platelet transfusion to reverse platelet inhibition remains unclear. Two systematic reviews and meta-analyses exist on the effect of early surgery in the trauma treatment of hip fractures in patients on APT before the injury, which is slightly different from the study on the impact of APT. Both studies revealed that early surgery for patients on APT who had hip fractures was associated with increased transfusion rates; however, a decreased mortality and length of hospital stay (32, 33). While some studies found no significant association between APT administration before major trauma injury and mortality or other outcomes, other studies reported increased mortality and rebleeding rates (8, 9, 31, 34). We could not examine single and double APT in this study; nonetheless, Ferraris et al. reported that patients with APT have significantly more comorbidities and worse outcomes with DAPT than SAPT in either case (35). Furthermore, examining whether aspirin or thienopyridines were administered was not possible but clinically there was no differential management of patients. One interesting study suggests that APT administration before trauma injury is associated with a decreased risk of lung dysfunction, multiple organ failure, and possibly death in high-risk patients with blunt trauma who received transfusions. This finding suggests that platelets are involved in the development of organ dysfunction, according to the author (36). As noted above, there is no consensus on how preinjury APT affects mortality and other outcomes of major trauma, not only a head injury. In the present study, there was no significant difference in mortality between the time point with or without APT before the injury. Considering the disadvantages of APT drug withdrawal, the results may support a treatment policy without discontinuing APT and delaying surgery.

This study had some limitations. First, our observations were limited to a relatively small population; a larger and more racially diverse data set should be the focus of future studies. Second, the groups were not randomized. Third, we could not distinguish single and dual platelet therapy or figure out medication compliance because data were obtained from an observational registry. Fourth, because of no prior development of transfusion protocols in this study, it was impossible to assess whether there was any arbitrary influence on the administration of RBCs, FFP, and PC. Therefore, a prospective study with a predefined protocol is desirable in the future.

## 5. Conclusion

Our findings suggest that patients administered AC have a higher risk of early mortality than patients not administered AC or AP despite the limitations of the study. In contrast, the mortality risk for patients administered AP remains unchanged. These results provide encouraging data regarding the approach to trauma care among patients receiving AP, despite the lack of reversible agents. However, further studies are needed to clarify the benefits and risks associated with AP.

## Data availability statement

The data that support the findings of this study are available from the corresponding author, upon reasonable request. Requests to access these datasets should be directed to the corresponding author HO, hideshi@gifu-u.ac.jp.

## Ethics statement

The Tohoku University Institutional Research Ethics Committee approved the use of the J-OCTET 2 (approval #2020-1-898, approved on January 15, 2021). Furthermore, the Gifu University Institutional Ethics Committee approved this study (approval #2022-141, approved on October 12, 2022). The Institutional Ethics Committees of Gifu University Graduate School of Medicine approved the substitution of an opt-out notice of informed consent from patients due to the retrospective nature of the study, whose design was based on computerized data with anonymous selection. Written informed consent for participation was not required for this study in accordance with the national legislation and the institutional requirements.

## Author contributions

FY, HO, RK, YKa, GY, YM, YKi, TF, TM, NK, TD, TY, SY, and SO: treatment of the patients. FY and RK: writing—original draft. FY, RK, and HO: writing—review and editing. TI: data management and analysis. All authors read and approved the final manuscript.
