# Corrigendum: Retrospective cohort study to determine the effect of preinjury antiplatelet or anticoagulant therapy on mortality in patients with major trauma

**DOI:** 10.3389/fmed.2023.1281829

**Published:** 2023-09-18

**Authors:** Fuminori Yamaji, Hideshi Okada, Ryo Kamidani, Yuki Kawasaki, Genki Yoshimura, Yosuke Mizuno, Yuichiro Kitagawa, Tetsuya Fukuta, Takuma Ishihara, Kodai Suzuki, Takahito Miyake, Norihide Kanda, Tomoaki Doi, Takahiro Yoshida, Shozo Yoshida, Shinji Ogura

**Affiliations:** ^1^Advanced Critical Care Center, Gifu University Hospital, Gifu, Japan; ^2^Abuse Prevention Center, Gifu University Graduate School of Medicine, Gifu, Japan; ^3^Innovative and Clinical Research Promotion Center, Gifu University Hospital, Gifu, Japan

**Keywords:** trauma, antiplatelet therapy, anticoagulant therapy, cohort study, J-OCTET 2, injury

In the published article, there was an error.

A correction has been made to Acknowledgments. This sentence previously stated:

“We thank Editage (www.editage.com) for English language editing.”

The corrected sentence appears below:

“We appreciated for J-OCTET 2 Investigators, who are Shigeki Kushimoto and Daisuke Kudo. Sendai: Tohoku University Graduate School of Medicine; Takayuki Ogura (Saiseikai Utsunomiya Hospital, Utsunomiya); Atsushi Shiraishi (Kameda Medical Center, Kamogawa); Akira Endo (Tokyo Medical and Dental University Hospital of Medicine, Tokyo); Yuya Yoshimura (National Defence Medical College, Tokorozawa); Kazuhiko Sekine (Tokyo Saiseikai Central Hospital, Tokyo); Takashi Tagami (Nippon Medical School Musashi Kosugi Hospital, Kawasaki); Kaori Ito (Teikyo University Hospital, Tokyo); Mineji Hayakawa (Hokkaido University Hospital, Sapporo); Toru Hifumi (St. Luke's International Hospital, Tokyo); Shunichiro Nakao (Osaka University Graduate School of Medicine, Osaka); and Akiyoshi Hagiwara (Niizashiki Chuo General Hospital, Saitama). We also thank Editage (www.editage.com) for English language editing.”

The authors apologize for this error and state that this does not change the scientific conclusions of the article in any way. The original article has been updated.

